# Pupil Size Prediction Techniques Based on Convolution Neural Network

**DOI:** 10.3390/s21154965

**Published:** 2021-07-21

**Authors:** Allen Jong-Woei Whang, Yi-Yung Chen, Wei-Chieh Tseng, Chih-Hsien Tsai, Yi-Ping Chao, Chieh-Hung Yen, Chun-Hsiu Liu, Xin Zhang

**Affiliations:** 1 Department of Electronic and Computer Engineering, National Taiwan University of Science and Technology, Taipei City 106335, Taiwan; whang@mail.ntust.edu.tw (A.J.-W.W.); D10502301@mail.ntust.edu.tw (W.-C.T.); M10802312@mail.ntust.edu.tw (X.Z.); 2 Graduate Institute of Color & Illumination Technology, National Taiwan University of Science and Technology, Taipei City 106335, Taiwan; 3 Graduate Institute of Electro-Optical Engineering, National Taiwan University of Science and Technology, Taipei City 106335, Taiwan; peter8411124146@gmail.com; 4 Graduate Institute of Biomedical Engineering, Chang Gung University, Taoyuan City 333323, Taiwan; yiping@mail.cgu.edu.tw (Y.-P.C.); chiehhungyen@gmail.com (C.-H.Y.); 5 Department of Computer Science and Information Engineering, Chang Gung University, Taoyuan City 333323, Taiwan; 6 Department of Neurology, Chang Gung Memorial Hospital at Linkou, Taoyuan City 333423, Taiwan; 7 Department of Ophthalmology, Chang Gung Memorial Hospital at Linkou, Taoyuan City 333423, Taiwan; cdeegg@gmail.com; 8 College of Medicine, Chang Gung University, Taoyuan City 333323, Taiwan

**Keywords:** biomedical imaging, computational intelligence, engineering in medicine and biology, machine learning

## Abstract

The size of one’s pupil can indicate one’s physical condition and mental state. When we search related papers about AI and the pupil, most studies focused on eye-tracking. This paper proposes an algorithm that can calculate pupil size based on a convolution neural network (CNN). Usually, the shape of the pupil is not round, and 50% of pupils can be calculated using ellipses as the best fitting shapes. This paper uses the major and minor axes of an ellipse to represent the size of pupils and uses the two parameters as the output of the network. Regarding the input of the network, the dataset is in video format (continuous frames). Taking each frame from the videos and using these to train the CNN model may cause overfitting since the images are too similar. This study used data augmentation and calculated the structural similarity to ensure that the images had a certain degree of difference to avoid this problem. For optimizing the network structure, this study compared the mean error with changes in the depth of the network and the field of view (FOV) of the convolution filter. The result shows that both deepening the network and widening the FOV of the convolution filter can reduce the mean error. According to the results, the mean error of the pupil length is 5.437% and the pupil area is 10.57%. It can operate in low-cost mobile embedded systems at 35 frames per second, demonstrating that low-cost designs can be used for pupil size prediction.

## 1. Introduction

The irises of lower vertebrates are intrinsically photosensitive, so a pupillary light reflex (PLR) does not need to be controlled by the brainstem. However, the PLR of higher vertebrates is governed by the brainstem [1]. The pupil size of the human eye is between 1.5 mm and 9 mm [2] and is controlled by the autonomic nerve. Thus, the optic nerve function of the central and peripheral nervous systems can be evaluated [3]. In clinical practice, the pupillary response to light stimuli evaluates the retina, optic nerve function, and brainstem [4]. PLR is essential in the diagnosis of eye diseases and nervous system research. PLR mainly measures the size (diameter or area) of the pupil [5], and the size of the pupil is controlled by the circular (sphincter) and radial muscles of the iris. The parasympathetic nervous system (PNS) innervates the circular muscle, and the sympathetic nervous system (SNS) controls the radial muscle [6]. Both PNS and SNS can be used as parameters to predict a patient’s physical condition. When a patient has inconsistent responses on both sides of the pupil, or the contraction response is different from ordinary people, it may be a sign of certain diseases [6,7,8,9,10,11,12,13].

Many studies have shown that, under the stimulation of red light and blue light, the pupil contraction in primary open-angle glaucoma (POAG) is either smaller than that in ordinary people or non-existent [7,8,13]. In addition to glaucoma and a series of retinopathy caused by diabetes, including patients with diabetic retinopathy (DR), non-proliferative diabetic retinopathy (NPDR), and proliferative diabetic retinopathy (PDR), the pupil response decreases as the disease worsens under the stimulation of a light source [6,9,13]. Retinopathy is usually accompanied by the reduced function of intrinsically photosensitive retinal ganglion cells (ipRGC). In addition to abnormal post illumination pupil response (PIPR), it can also cause the dysregulation of circadian rhythms [9,13]. In addition to the lesions mentioned above, conditions caused by abnormalities of the central nervous system can also affect the pupil. For example, the pupils of patients under general anesthesia and suffering from Horner syndrome will respond differently to light source stimulation than those of ordinary people [10,11,12]. Pupil size is also very helpful in psychology; the size of the pupil changes with mood. The pupil dilates when a subject is in a pleasant mood; otherwise, it constricts [14,15]. In clinical practice, the size of the pupil is predicted by the experience of the medical staff. The behavior will not be unified since it depends on the subjective consciousness or the degree of fatigue among medical staff [16]. Medical equipment for pupil measurement is expensive and must rely on the cooperation of patients, which is inconvenient for medical staff.

For these reasons, this paper suggests that there must be a way to quantify pupil size, which is convenient and has a low cost. In the past, most studies have focused on pupil centering or eye-tracking [17,18,19,20,21,22,23]. There are few studies on pupil size. Concerning the study of pupil size, for example, Garcia et al., used OpenCV to preprocess an image and then used the Otsu threshold and contour detection to complete pupil size detection [16]. De Souza et al., proposed using two complementary independent algorithms. They then used the results of these two separate algorithms to infer the center of the pupil and looked outward to the edge to complete the pupil size prediction [24]. De Santis et al., used the level set theory to realize fully automatic segmentation and then used this result to predict pupil size [25]. Thasina Tabashum et al., used the Kalman Filter to develop a real-time prototype that can simultaneously extract pupil size over time and enable adjustment frame by frame [26]. S Navaneethan et al., proposed a human eye pupil detection system to quickly recognize and diagnose the human eye pupil area. Double threshold, logical OR, morphological closing, and average black pixel density modules are involved in the proposed solution [27]. These studies about pupil size most used rule-based algorithms. Additionally, Taehyung Kim et al., used a convolutional neural network-based semantic segmentation method for accurate pupil detection [28] that mixes the rule-based algorithm with an AI-based program. Deep learning is a type of artificial intelligence (AI). In terms of AI acceptance, 84.2% of healthcare workers agree that AI can assist the imaging and pathology department, and 76.3% of non-healthcare workers agree that AI is helpful [29]. Therefore, this paper proposes an algorithm based on deep learning that allows real-time calculations in a low-cost mobile embedded system. The research will help solve clinical problems in pupil measurement, including the fact that pupil size is unable to be quantified in real-time, involves inconvenient operation, and is often expensive. The main contributions of this paper include:

- We have proposed pupil size detection based on a convolution neural network that allows real-time calculation in a low-cost mobile embedded system.
- We have evaluated the performance of the proposed approach with multiple realistic datasets for optimizing the structure.

## 2. Materials and Methods

### 2.1. Dataset

This study chose three different datasets, labeled pupils, from the wild (LPW), CASIA-Iris, and Świrski datasets. The purpose was to prevent the training of the only dataset, which would create overfitting. In addition, we selected images from datasets, except for eye images with complete pupils for training, and struck off any photographs that showed the pupil partially covered. Additionally, the training data added complex pupil images, such as those which included glasses and makeup. Those taken outdoor also increased the challenge of the model.

#### 2.1.1. Labeled Pupils in the Wild Dataset

**Figure 1:**
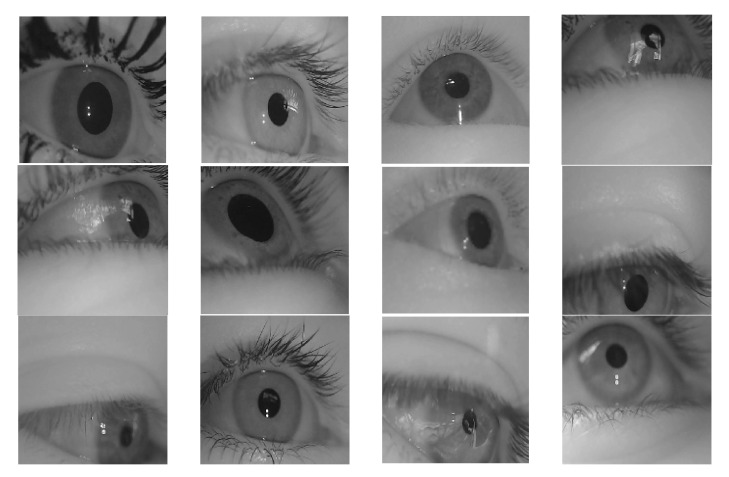
The sample images of the LPW dataset.

**Figure 2:**
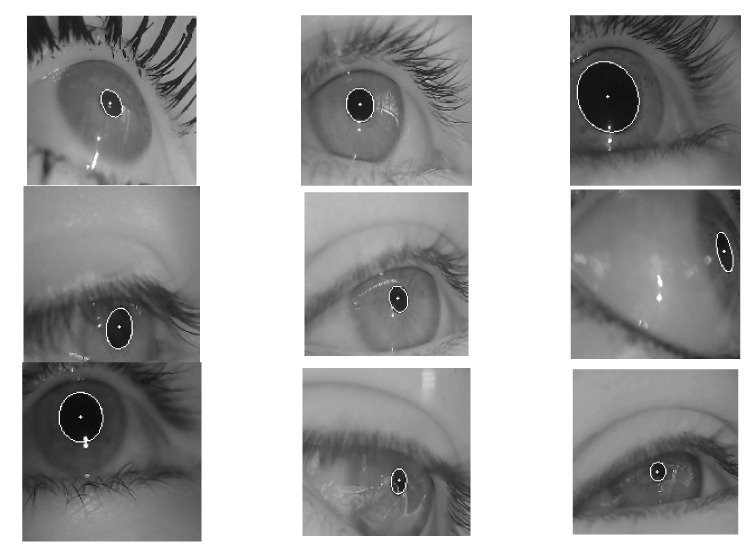
The result of the LPW dataset ellipse fitting.

The dataset used in this study is labeled as pupils in the wild (LPW) and was provided by Tonsen et al. [30]. The first dataset had nine different conditions for high-quality eye images. It provided a label for the center of the pupil, which strengthened the challenge and accuracy of our model. The images of this dataset are shown in Figure 1 and Figure 2 with ellipse fitting.

LPW was provided in the form of videos, so this study needed to extract the images from the video in an appropriate sampling frequency. The videos were continuous frames, so the similarity of adjacent photos was high. The high similarity could easily cause over-training. Therefore, this study performed a structural similarity analysis to ensure that the sampling frequency was not too high. The calculation method of the structural similarity index is as follows:

(1) $$SSIM\left(x,y\right)=\frac{\left(2\mu_{x}\mu_{y}+C_{1}\right)\left(2\sigma_{xy}+C_{2}\right)}{\left(\mu_{x}^{2}+\mu_{y}^{2}+C_{1}\right)\left(\sigma_{x}^{2}+\sigma_{y}^{2}+C_{2}\right)}$$

where the $\mu_{x}$ and $\mu_{y}$ are mean values, $\sigma_{x}$ and $\sigma_{y}$ are standard deviations, $\sigma_{xy}$ is the covariance, and $C_{1}$ and $C_{2}$ are constants used to control the overall stability. If the structural similarity index is 1, the two images do not have a difference.

This study compared the structural similarity index with different intervals of 10, 15, and 20 frames, and the average value of SSIM is shown in Table 1. Finally, this study chose the interval of 15 frames, in which the average value of the structural similarity index was closest to 0.7992 [31].

#### 2.1.2. CASIA-IrisV4-Thousand Dataset

**Figure 3:**
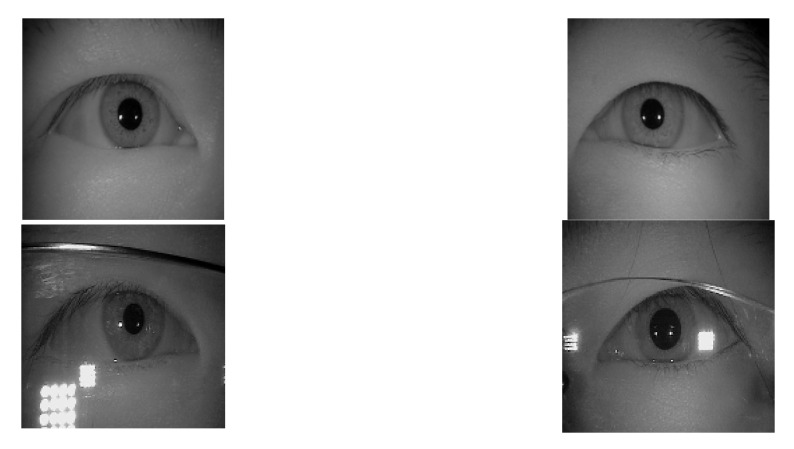
The sample images of the CASIA-IrisV4-Thousand dataset.

**Figure 4:**
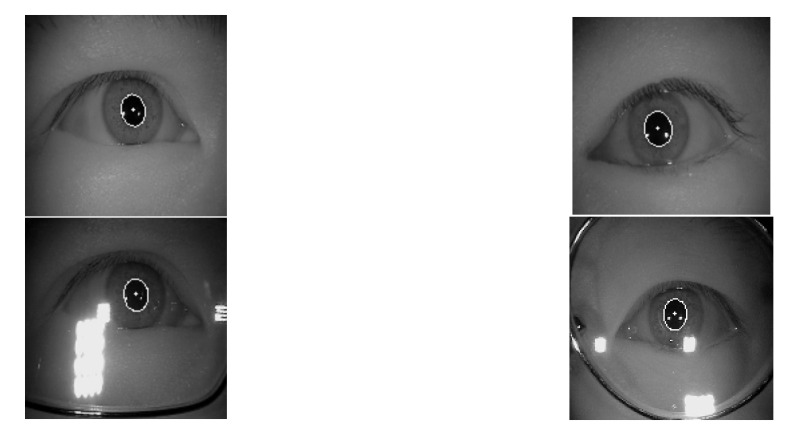
The result of the CASIA-IrisV4-Thousand dataset ellipse fitting.

The LPW dataset was taken with the eye tracker located on the edge of glasses, so the oblique image was obtained. In addition, since the pictures of LPW are from videos, photos from the same person may be split into training and test datasets, and we need other datasets to reduce the impact. Therefore, for the second dataset, we added CASIA-Iris-Thousand. These data come from the Chinese Academy of Sciences’ Institute of Automation (CASIA), a subset of CASIA-IrisV4 [32]. Additionally, CASIA-Iris-Thousand used an IKEMB-100 camera produced by IrisKing, which captured 1000 subjects to obtain 20,000 high-quality positive iris images. The images of this dataset are shown in Figure 3 and Figure 4 with ellipse fitting.

#### 2.1.3. ŚWirski Dataset

**Figure 5:**
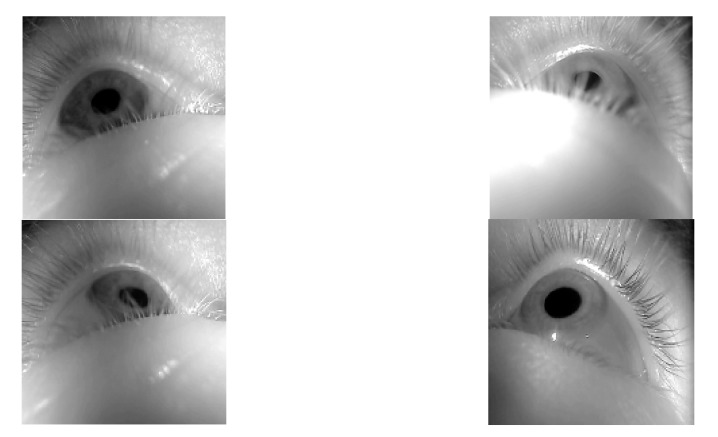
The sample images of the Świrski dataset.

**Figure 6:**
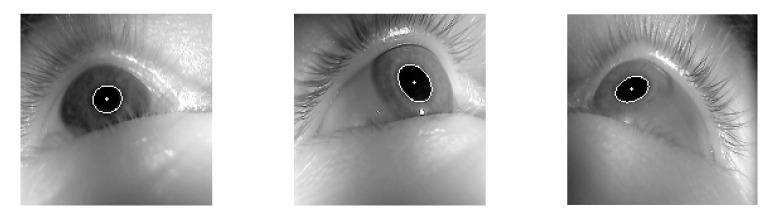
The result of the Świrski dataset ellipse fitting.

Since the LPW and CASIA-IrisV4-Thousand datasets were pretty different, we chose to add a third dataset. This dataset was provided by Lech Świrski et al. [33]. These data contained four datasets as single images. As with the LPW dataset, both were shot obliquely with the eye tracker on the glasses. Similar to the CASIA-IrisV4-Thousand dataset, the eye position was not exaggerated. It was appropriate to join and neutralize the two datasets. The images of this dataset are shown in Figure 5 and Figure 6 with ellipse fitting.

#### 2.1.4. Preprocess Details

In addition to the similar structure index, data augmentation was also applied to ensure that the network could learn more styles during training. For data balance, the dataset will use data augmentation to solve different amounts of data. For data augmentation, this study used scales between 0.7 and 1.3 to produce different pupil sizes. The data were also shifted 10% in each axis to allow the pupil edge to be at the image edge or even beyond the image. Moreover, we used a rotation of ±30° and flipped vertically and horizontally to cope with various situations. The items we used in data augmentation will not change the image’s label to simplify the data procession. After that, we have 60,000 images, and the size is 152 by 152 pixels. All of them could enhance the richness of the dataset. Finally, the dataset was split into the training set and the testing set in a ratio of 7:3, which would be shuffled before data split.

Of the three datasets, some only provided the label of the pupil center, so we needed a new label for length. In both dark and light environments, human pupils have an average non-circularity of 0.0166. The ellipse’s fitting abilities in both the dark and light were 59.6% and 47.7%, respectively, so the fitting shape was set to ellipse [34]. The ground truth of the network was calculated by the pixels on the major and minor axes of the ellipse fitted by a direct least-squares method [35].

### 2.2. Method and Network Structure

**Figure 7:**
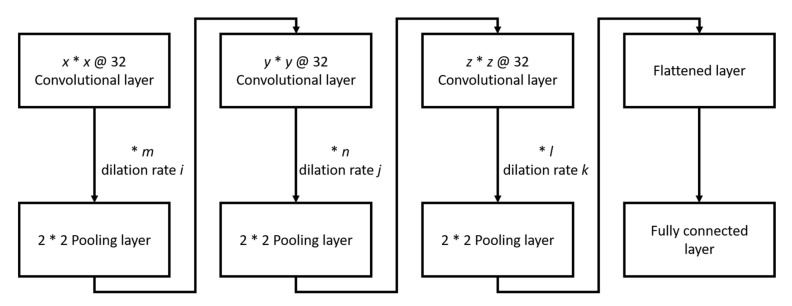
Network structure.

Before training, we divided the dataset into two parts. One was the training set, and the other was used for testing the models after training. As this study was limited by hardware that could not put all of the training data into the network simultaneously, the training set was split into three parts and loaded into the network in stages. That ensured that the training data in each stage were unknown. In addition to different training data, the learning rate of each step was also different. The learning rate was dropped from 1 × 10^−3^ to 1 × 10^−5^ at each step, and the batch size was 128. Every stage-trained for 100 epochs and used Adam as the optimizer. The network structure is shown in Figure 7, and the * symbol means multiplication to avoid confusion with the letter x.

The network parameters are shown in Table 2. Concerning the symbols used, x, y, and z of K_(x,y,z)_ are the size of the three-dimensional filters, and the amount of filters in each layer is fixed at 32. Besides, m, n, and l of C_(m,n,l)_ are the number of convolution layers before the pooling layer, and i, j, and k of D(i, j, k) are the dilation rates of the convolution layer. After the convolution layers, one 2D max-pooling layer reduces noise, and the size is two by two.

**Figure 8:**
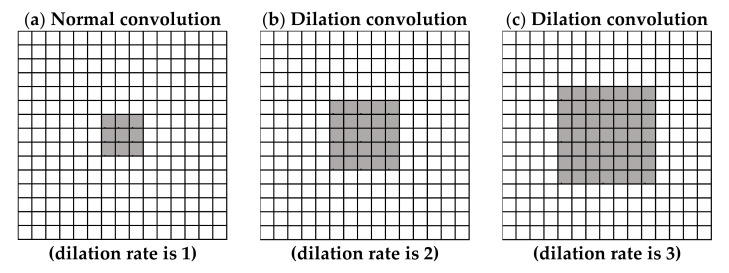
Diagram convolution with a 3×3 filter. The black dot is the position where the convolution occurs, and the gray area is the FOV of the filter. The dilation rates are (**a**) = 1, (**b**) = 2, and (**c**) = 3.

The purpose of this study was to achieve a real-time prediction in a low-cost mobile embedded system, so the number of parameters could not be too large. A large model, or too many parameters, would not achieve the required speed using a low-cost mobile embedded system, so this study used dilation convolution to accomplish this purpose. The dilation convolution is shown in Figure 8.

The dilation convolution can enlarge the FOV of the filter without increasing the number of parameters. Widening the FOV can enable a broader range of information to be accepted [36]. Additionally, it can effectively prevent the misjudgment of the network caused by some similar features. This study also compared the network models with the same FOV between the regular filter and the dilated convolution to understand the effect of the decrease in the number of parameters by dilation convolution. Therefore, in addition to three different depth networks in Table 2, there were also three types with varying convolution structures.

## 3. Results

This study used the test datasets for testing the networks and used the mean error as metrics, which are defined as follows:

(2) $$\mathrm{mean}\;\mathrm{error}=\frac{1}{n}\sum \frac{\left|\mathrm{prediction}(\mathrm{pix})-\mathrm{ground}\;\mathrm{truth}(\mathrm{pix})\right|}{\mathrm{ground}\;\mathrm{truth}(\mathrm{pix})}\times 100\%$$

As the mean error simultaneously calculates the error of the major and minor axes, *n* is equal to twice the total number of images. The results of the test are shown in Table 3:

The mean error of length of all networks is mostly within 6%, and the best model even reaches 2.660%.

**Figure 9:**
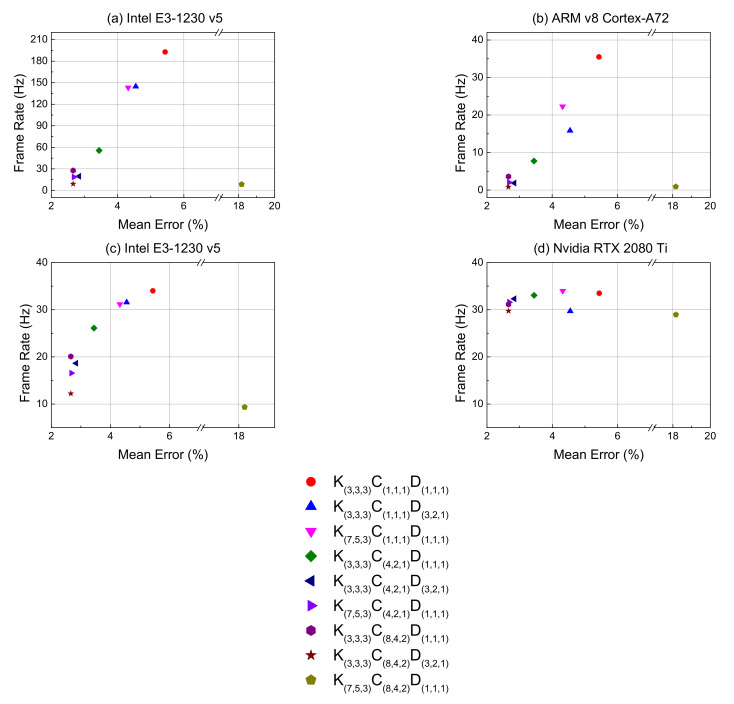
The relationship between frame rate and mean error under different hardware conditions for each network. (**a**,**b**) use the TensorFlow Lite model; (**c**,**d**) use the original Keras model.

For testing the speed on a low-cost mobile embedded system, this study converted the original Keras model to the TensorFlow Lite model, which can be used for Raspberry Pi to (a) Intel E3-1230 v5 and (b) ARM v8 Cortex-A72 (Raspberry Pi 4 Model B). This study also tested the speed on the original Keras model of (c) Intel E3-1230 v5 and (d) NVIDIA RTX 2080 Ti. The results are shown in Figure 9.

On the (a) Intel E3-1230 v5 and (b) ARM v8 Cortex-A72, the highest speeds were around 55 FPS and 35 FPS. On the (c) Intel E3-1230 v5 and (d) NVIDIA RTX 2080 Ti, the highest speed was around 31 FPS and 31 FPS.

This study considered that the real-time calculation was faster than 30 FPS [37] in the low-cost mobile embedded system, so the model with the lowest mean error was K_(3,3,3)_C_(1,1,1)_D_(1,1,1)_. Therefore, this study suggests using K_(3,3,3)_C_(1,1,1)_D_(1,1,1)_ as the final model for this study. Under the same conditions, we recommend choosing K_(3,3,3)_C_(4,2,1)_D_(1,1,1)_ and K_(3,3,3)_C_(8,4,2)_D_(3,2,1)_ for (c) Intel E3-1230 v5 and (d) NVIDIA RTX 2080 Ti as the suggested model for different hardware.

## 4. Discussion

### 4.1. Model Evaluation

Although the model can reach a maximum of 35 FPS in a low-cost mobile embedded system, its mean error is too high. In addition, the model with the lowest mean error of 2.660% is too slow. When the pupil size changes, it may not detect all changes in pupil size in real-time because the calculation speed is insufficient. As such, this study used 30 FPS as the condition.

In addition to the length of major and minor axes, the area can also express the pupil size. The equation is as follows:

(3) Ellipse Area=a×b×π

where *a* is the semi-major axis, and *b* is the semi-minor axis. In this study, the mean error of the length is not counted separately for the major and minor axes, so the ellipse area equation is used with the mean error of length. In the recommended model, the mean error of the length and area are 5.437% and 10.57%, respectively. Besides, the comparison with previous research [16] is shown in Table 4.

### 4.2. Feature Map Visualization

**Figure 10:**
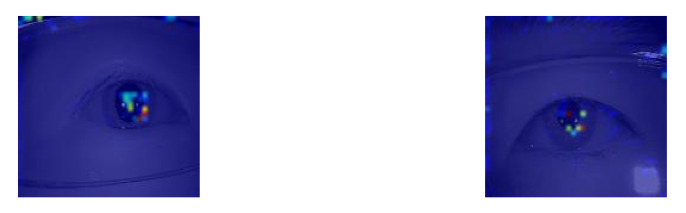
The feature map visualization.

This paper uses the GRAD-CAM [38] method to calculate its weight (w) by backpropagation to confirm the relevance of the feature map and the parameters. It can be observed from the image that the area of interest of the network architecture is the pupil area, as shown in Figure 10.

### 4.3. Model Speed Trend

**Figure 11:**
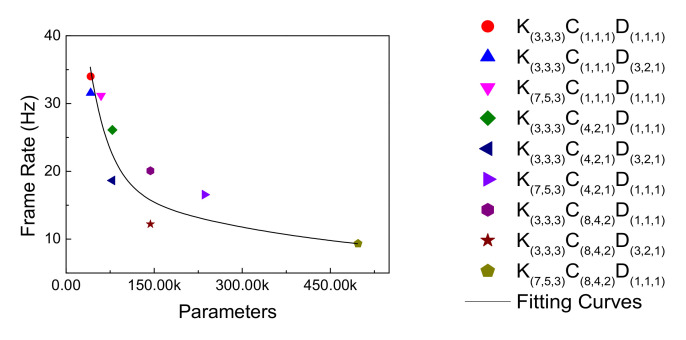
Trend curves of frame rate and parameters.

Figure 11 shows the relationship between the frame rate of each model and its number of parameters. According to the results, the frame rate decreases as the number of parameters increases, and its fitting curve shows an exponential trend. The coefficient of determination (R^2^) of the fitting curve in Figure 11 is 0.8168, and it can be concluded that the frame rate and the number of parameters are highly correlated. Therefore, this study tried to reduce the number of parameters by dilation convolution, which is conducive to improving the network’s speed.

### 4.4. Model Comparison

**Figure 12:**
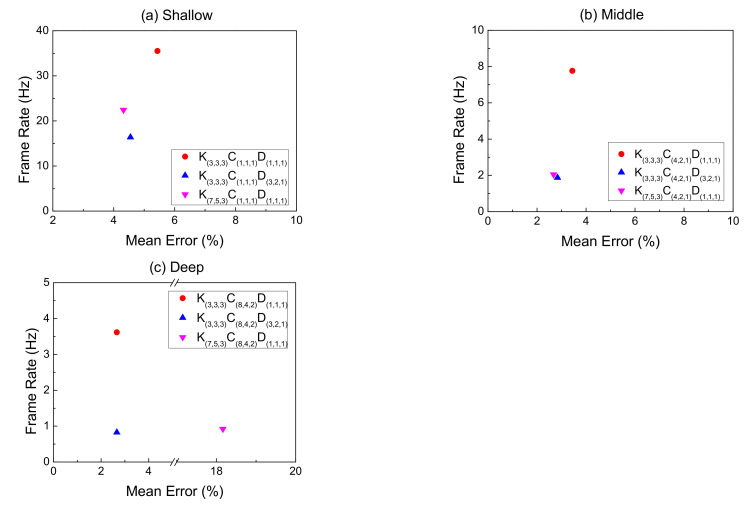
The effect of the filter’s FOV changes on speed and mean error with (**a**) Shallow structure, (**b**) Middle structure, (**c**) Deep structure.

Figure 12 shows the effect of changing the convolution filter at the same depth. Both Type II and Type III can effectively reduce the mean error in most cases. However, the speed decreases. The declining rate on Type II models is more considerable at the shallower structure than Type III, but the difference between the two becomes smaller in the middle, as shown in Figure 12a,b. In Figure 12c, the Type III model may have too many useless features leading to errors in judgment, although the convolution filter has a larger FOV. Therefore, the overall mean error is higher than Type II.

### 4.5. Model Revision

From the experimental results, this study found that in the Type II and Type I models with the same parameters, while the mean error of Type II is significantly reduced the calculation speed is slightly reduced. That is probably due to the fact that the Type II model has a larger FOV than Type I. Although the part with more FOV seems to skip calculation when performing convolution operations, the convolution kernel size is not reduced. The neglected part can be regarded as a fixed-zero weight calculation, so the part will not be trained, and the number of parameters will not increase. However, complementing the part where the weight of the filter is regarded as zero will create too many parameters. The larger FOV of the convolution kernel will decrease operating efficiency, resulting in a slower speed. In addition to wasting performance, it is also easy to affect the prediction because of some useless features.

This study conducted another experiment to account for the suggestion that the number of parameters causes the mean error to rise. This study tried to reduce the number of parameters in the deepest network of Type III. As the depth of the network was fixed, we adjusted the number of filters in each layer. We then changed the 32 filters to use between 4 and 10 filters, and the mean error was reduced to within 10%. However, if more than 11 convolution filters were used, the mean error would not decrease significantly. According to the results, the mean error will reduce by lowering the number of parameters, as expected. Therefore, due to too many network parameters, the deepest network of Type III will cause the mean error to fail to achieve the desired result, resulting in serious misjudgment.

## 5. Conclusions

This paper proposes an algorithm that can calculate pupil size that is based on the CNN model. This study compared the mean error with different network depths and FOV of the convolution filter for optimizing the structure. According to the results, both deepening the network and widening the FOV can effectively reduce the mean error. However, both will reduce speed. For the ARM v8 Cortex-A72 (RPi 4B), we recommend the model be used at 35 FPS. The mean error of the pupil length is 5.437%, and the pupil area is 10.57%. The model can calculate, in real-time, pupil size in a low-cost mobile system. It will help doctors and nurses to obtain quantified results. This study also provides models with lower mean error under different hardware conditions and respective improvements in calculation speeds.

We will make an in-depth study to optimize the neural network structure of the system to reduce the computational complexity and improve pupil detection speed in future works. For the algorithm to identify pupils in different fields, the richness of the dataset will be increased to enhance the generalization of real situations. We also need to consider that we split the training and test sets after the data augmentation may allow the model to peek at the test data during the training process and cause deviation. Besides, arranging clinical experiments to compare with the results of machines and doctors is also necessary.

## Figures and Tables

**Table 1:** The average value of SSIM at the different intervals of sampling.

Interval of Sampling	Average Value
10	0.8406
15	0.8090
20	0.7781

**Table 2:** Network parameters.

		Comparison Type		
		Type I	Type II	Type III
Network depth	Shallow	K_(3,3,3)_C_(1,1,1)_D_(1,1,1)_	K_(3,3,3)_C_(1,1,1)_D_(3,2,1)_	K_(7,5,3)_C_(1,1,1)_D_(1,1,1)_
	Middle	K_(3,3,3)_C_(4,2,1)_D_(1,1,1)_	K_(3,3,3)_C_(4,2,1)_D_(3,2,1)_	K_(7,5,3)_C_(4,2,1)_D_(1,1,1)_
	Deep	K_(3,3,3)_C_(8,4,2)_D_(1,1,1)_	K_(3,3,3)_C_(8,4,2)_D_(3,2,1)_	K_(7,5,3)_C_(8,4,2)_D_(1,1,1)_

Type I uses regular convolution; Type II uses dilation convolution; Type III uses regular convolution. The filter size has the same FOV as Type II.

**Table 3:** Mean error of each network.

		Comparison Type		
		Type I	Type II	Type III
Network depth	Shallow	5.437%	4.549%	4.321%
	Middle	3.442%	2.838%	2.677%
	Deep	2.662%	2.660%	18.165%

**Table 4:** The comparison with previous research.

	The Recommended Model	The Previous Research
Mean Error	5.437%	6.587%

## Data Availability

The data of LPW was obtained from Tonsen, M., etc., and are available from http://doi.org//10.1145/2857491.2857520 with openly available. The data of CASIA-IrisV4 was obtained from the Chinese Academy of Sciences’ Institute of Automation (CASIA) and available from http://www.cbsr.ia.ac.cn/china/Iris%20Databases%20CH.asp with permission of the Center for Biometrics and Security Research (CBSR). The data of Świrski was obtained from Świrski, L., etc., and are available from http://www.cl.cam.ac.uk/research/rainbow/projects/pupiltracking/ with openly available.

## References

[1] Xue T., Do M.T.H., Riccio A., Jiang Z., Hsieh J., Wang H.C., Merbs S.L., Welsbie D.S., Yoshioka T., Weissgerber P. (2011). Melanopsin signalling in mammalian iris and retina. Nat. Cell Biol..

[2] Kret M.E., Sjak-Shie E.E. (2019). Preprocessing pupil size data: Guidelines and code. Behav. Res. Methods.

[3] Bitsios P., Prettyman R., Szabadi E. (1996). Changes in Autonomic Function with Age: A Study of Pupillary Kinetics in Healthy Young and Old People. Age Ageing.

[4] Canver M.C., Canver A.C., Revere K.E., Amado D., Bennett J., Chung D.C. (2014). Novel mathematical algorithm for pupillometric data analysis. Comput. Methods Programs Biomed..

[5] Lu W., Tan J., Zhang K., Lei B. (2008). Computerized mouse pupil size measurement for pupillary light reflex analysis. Comput. Methods Programs Biomed..

[6] Jain M., Devan S., Jaisankar D., Swaminathan G., Pardhan S., Raman R. (2018). Pupillary Abnormalities with Varying Severity of Diabetic Retinopathy. Sci. Rep..

[7] Rukmini A.V., Milea D., Baskaran M., How A.C., Perera S.A., Aung T., Gooley J.J. (2015). Pupillary Responses to High-Irradiance Blue Light Correlate with Glaucoma Severity. Ophthalmology.

[8] Chang D.S., Boland M.V., Arora K.S., Supakontanasan W., Chen B.B., Friedman D.S. (2013). Symmetry of the Pupillary Light Reflex and Its Relationship to Retinal Nerve Fiber Layer Thickness and Visual Field Defect. Investig. Opthalmol. Vis. Sci..

[9] Reutrakul S., Crowley S.J., Park J.C., Chau F.Y., Priyadarshini M., Hanlon E.C., Danielson K.K., Gerber B.S., Baynard T., Yeh J.J. (2020). Relationship between Intrinsically Photosensitive Ganglion Cell Function and Circadian Regulation in Diabetic Retinopathy. Sci. Rep..

[10] Larson M.D., Behrends M. (2015). Behrends, and Analgesia, Portable infrared pupillometry: A review. Anesth. Analg..

[11] O’Neill W., Trick K. (2001). The narcoleptic cognitive pupillary response. IEEE Trans. Biomed. Eng..

[12] Yoo Y.J., Yang H.K., Hwang J.-M. (2017). Efficacy of digital pupillometry for diagnosis of Horner syndrome. PLoS ONE.

[13] Adhikari P., Zele A.J., Feigl B. (2015). The Post-Illumination Pupil Response (PIPR). Investig. Opthalmol. Vis. Sci..

[14] Mitz A.R., Chacko R.V., Putnam P.T., Rudebeck P.H., Murray E.A. (2017). Using pupil size and heart rate to infer affective states during behavioral neurophysiology and neuropsychology experiments. J. Neurosci. Methods.

[15] Wang C.-A., Baird T., Huang J., Coutinho J.D., Brien D.C., Munoz D.P. (2018). Arousal Effects on Pupil Size, Heart Rate, and Skin Conductance in an Emotional Face Task. Front. Neurol..

[16] Garcia R.G., Avendano G.O., Agdeppa D.B.F., Castillo K.J., Go N.R.S., Mesina M.A. Automated Pupillometer Using Edge Detection in OpenCV for Pupil Size and Reactivity Assessment. Proceedings of the 2019 3rd International Conference on Imaging, Signal Processing and Communication (ICISPC).

[17] Fuhl W., Rosenstiel W., Kasneci E. (2019). 500,000 Images Closer to Eyelid and Pupil Segmentation. Transactions on Petri Nets and Other Models of Concurrency XV.

[18] Miron C., Pasarica A., Bozomitu R.G., Manta V., Timofte R., Ciucu R. Efficient Pupil Detection with a Convolutional Neural Network. Proceedings of the 2019 E-Health and Bioengineering Conference (EHB).

[19] Vera-Olmos F., Pardo E., Melero H., Malpica N. (2018). DeepEye: Deep convolutional network for pupil detection in real environments. Integr. Comput. Eng..

[20] Yiu Y.-H., Aboulatta M., Raiser T., Ophey L., Flanagin V.L., zu Eulenburg P., Ahmadi S.-A. (2019). DeepVOG: Open-source pupil segmentation and gaze estimation in neuroscience using deep learning. J. Neurosci. Methods.

[21] Fuhl W., Santini T., Kasneci G., Rosenstiel W., Kasneci E. (2017). Pupilnet v2.0: Convolutional neural networks for cpu based real time robust pupil detection. arXiv.

[22] Vera-Olmos F.J., Malpica N. (2017). Deconvolutional Neural Network for Pupil Detection in Real-World Environments. Transactions on Petri Nets and Other Models of Concurrency XV.

[23] Fuhl W., Santini T.C., Kübler T., Kasneci E. (2016). Else: Ellipse selection for robust pupil detection in real-world environments. Proceedings of the Ninth Biennial ACM Symposium on Eye Tracking Research & Applications.

[24] De Souza J.K.S., Pinto M.A.D.S., Vieira P.G., Baron J., Tierra-Criollo C.J. (2013). An open-source, FireWire camera-based, Labview-controlled image acquisition system for automated, dynamic pupillometry and blink detection. Comput. Methods Programs Biomed..

[25] De Santis A., Iacoviello D. (2006). Optimal segmentation of pupillometric images for estimating pupil shape parameters. Comput. Methods Programs Biomed..

[26] Tabashum T., Zaffer A., Yousefzai R., Colletta K., Jost M.B., Park Y., Chawla J., Gaynes B., Albert M.V., Xiao T. (2021). Detection of Parkinson’s Disease Through Automated Pupil Tracking of the Post-illumination Pupillary Response. Front. Med..

[27] Navaneethan S., Nandhagopal N. (2021). RE-PUPIL: Resource efficient pupil detection system using the technique of average black pixel density. Sādhanā.

[28] Kim T., Lee E.C. (2020). Experimental Verification of Objective Visual Fatigue Measurement Based on Accurate Pupil Detection of Infrared Eye Image and Multi-Feature Analysis. Sensors.

[29] Xiang Y., Zhao L., Liu Z., Wu X., Chen J., Long E., Lin D., Zhu Y., Chen C., Lin Z. (2020). Implementation of artificial intelligence in medicine: Status analysis and development suggestions. Artif. Intell. Med..

[30] Tonsen M., Zhang X., Sugano Y., Bulling A. (2016). Labelled pupils in the wild: A dataset for studying pupil detection in unconstrained environments. Proceedings of the Ninth Biennial ACM Symposium on Eye Tracking Research & Applications.

[31] Zhang J., Liang X., Wang M., Yang L., Zhuo L. (2020). Coarse-to-fine object detection in unmanned aerial vehicle imagery using lightweight convolutional neural network and deep motion saliency. Neurocomputing.

[32] Portions of the Research in This Paper Use the CASIA-IrisV3 Collected by the Chinese Academy of Sciences’ Institute of Automation (CASIA) and a Reference to CASIA Iris Image Database. http://biometrics.idealtest.org/.

[33] Świrski L., Bulling A., Dodgson N. (2012). Robust real-time pupil tracking in highly off-axis images. Proceedings of the Symposium on Applied Computing.

[34] Wyatt H.J. (1995). The form of the human pupil. Vis. Res..

[35] FitzGibbon A., Pilu M., Fisher R. (1999). Direct least square fitting of ellipses. IEEE Trans. Pattern Anal. Mach. Intell..

[36] Yu F., Koltun V. (2015). Multi-scale context aggregation by dilated convolutions. arXiv.

[37] Rickmann A., Waizel M., Kazerounian S., Szurman P., Wilhelm H., Boden K.T. (2016). Digital Pupillometry in Normal Subjects. Neuro Ophthalmol..

[38] Selvaraju R.R., Cogswell M., Das A., Vedantam R., Parikh D., Batra D. Grad-CAM: Visual Explanations from Deep Networks via Gradient-Based Localization. Proceedings of the 2017 IEEE International Conference on Computer Vision (ICCV).

